# Invariance of the Trait Emotional Intelligence Construct Across Clinical Populations and Sociodemographic Variables

**DOI:** 10.3389/fpsyg.2022.796057

**Published:** 2022-04-01

**Authors:** Pablo Alejandro Pérez-Díaz, Denisse Manrique-Millones, María García-Gómez, Maria Isabel Vásquez-Suyo, Rosa Millones-Rivalles, Nataly Fernández-Ríos, Juan-Carlos Pérez-González, K. V. Petrides

**Affiliations:** ^1^Institute of Psychology, Sede Puerto Montt, Austral University of Chile, Puerto Montt, Chile; ^2^Carrera de Psicología, Universidad Científica del Sur, Lima, Peru; ^3^University Institute for Social Development and Sustainability (INDESS), Universidad de Cádiz, Cádiz, Spain; ^4^Psychiatric Academic Department, National University of San Marcos, Lima, Peru; ^5^Facultad de Educación y Psicología, Universidad Marcelino Champagnat, Lima, Peru; ^6^Facultad de Medicina, Universidad Nacional de San Agustín de Arequipa, Arequipa, Peru; ^7^Emotional Education Laboratory (EDUEMO Lab), Faculty of Education, National University of Distance Education (UNED), Madrid, Spain; ^8^Department of Clinical, Educational and Health Psychology, University College London, London, United Kingdom; ^9^London Psychometric Laboratory, London, United Kingdom

**Keywords:** trait emotional intelligence, cross-cultural, clinical population, measurement invariance, sociodemographic variables

## Abstract

Recent research has shown that cultural, linguistic, and sociodemographic peculiarities influence the measurement of trait emotional intelligence (trait EI). Assessing trait EI in different populations fosters cross-cultural research and expands the construct’s nomological network. In mental health, the trait EI of clinical populations has been scarcely researched. Accordingly, the present study examined the relationship between trait EI and key sociodemographic variables on Trait Emotional Intelligence Questionnaire (TEIQue-SF) datasets with mental healthcare patients from three different Spanish-speaking countries. Collectively, these datasets comprised 528 participants, 23% from Chile (120), 28% from Peru (150), and 49% from Spain (258). The sociodemographic variables we used for trait EI comparisons were gender, age, educational level, civil status, and occupational status. Analyses involved Multigroup Exploratory Structural Equation Modelling (to test measurement invariance) and analysis of covariance (ANCOVA). Our results revealed significant between-country differences in trait EI across the studied sociodemographic variables and interactions between these variables. Measurement invariance across the datasets was attained up to the scalar level regarding gender and education (i.e., strong invariance), although analyses on age, civil status, and occupation displayed non-invariance. The resultant psychometric evidence supports the suitability of the TEIQue-SF for the accurate cross-cultural assessment of trait EI in mental health settings. It also highlights the importance of incorporating trait EI into extant psychotherapeutic frameworks to enhance non-pharmacological treatment efficacy.

## Introduction

### Trait Emotional Intelligence Theory, Factor-Structure, and Measures

Trait EI is a personality-based conceptualisation of EI that is consistent with established models of Differential Psychology and has shown exceptionally strong evidence of construct validity (Petrides et al., 2016). Trait EI essentially concerns people’s perceptions of their emotional and social effectiveness (Petrides et al., 2007c; Van der Linden et al., 2017). The TEIQue was explicitly developed as the operationalisation vehicle for trait EI theory, and it is the instrument that comprehensively covers the sampling domain of the construct (Petrides et al., 2007a). The factor structure of the questionnaire comprises global trait EI at its apex, four interrelated factors in the middle and fifteen narrow facets at the bottom characterising the general attribute (Petrides, 2009). Short forms, like the TEIQue-SF, were intended as valid measures of the global trait EI factor, although they also allow accessing the four-factor structure, whereas full trait EI forms allow for facets descriptions as the last unit of the psychometric description of the construct (Cooper and Petrides, 2010).

The four-factor basis of the TEIQue comprises Wellbeing, Self-control, Emotionality, and Sociability (Petrides, 2009). Moreover, there is a general trait EI factor accounting for global emotion-related variability, namely, global trait EI. According to Petrides (2009), individuals who are generally better adapted feel positive, happy, and fulfilled, will score high on Wellbeing; those with strong determination and a healthy degree of control over their urges and desires will score high on Self-control; those who see themselves as emotionally capable and are in touch with their own and other’s people feelings will score high on Emotionality, and those who believe they are socially competent, good listeners, and can communicate assertively with people from heterogeneous backgrounds will score high on Sociability.

### Trait Emotional Intelligence as a Predictor of Wellbeing Measures in Mostly Healthy Samples

In contrast to most other EI measures, the suite of Trait Emotional Intelligence Questionnaire (TEIQue) assessments has fully developed theoretical foundations and nomological networks, spanned associations with important health outcomes (for reviews, see Martins et al., 2010; Batselé et al., 2019; and Sarrionandia and Mikolajczak, 2020), academic performance (MacCann et al., 2020), job satisfaction (Li et al., 2018; Gong et al., 2020), life satisfaction and subjective happiness (Stamatopoulou et al., 2016), stress management (Martínez-Monteagudo et al., 2019), and other primary psychological variables (see Andrei et al., 2016; for a review). Moreover, the literature has shown consistent incremental effects beyond the Big Five and cognate variables in the prediction of critical clinical criteria (Siegling et al., 2015b; Andrei et al., 2016), both with full and the short form of the trait EI questionnaire.

Different researchers have reported a negative correlation between trait EI and depressive, anxious, phobic, and obsessive symptoms (for a review, Zeidner et al., 2012). For instance, Mikolajczak et al. (2009) reported that trait EI moderated the impact of laboratory-induced stress on mood change, meaning that higher trait EI scores were significantly associated with less mood deterioration. The authors suggested that screening populations with trait EI measures is more efficient than assessing them on generic personality constructs, such as the Big Five, as trait EI provides more comprehensive coverage of emotion-related characteristics, it has demonstrated to negatively predict, over the Big Five, multiple clinical criteria, such as depression, stress, anxiety, and to positively predict outcomes, i.e., motivation, satisfaction with life (Siegling et al., 2015b), and needs fulfilment (i.e., psychological needs considered critical nutrients for optimal functioning, Barberis et al., 2018). Similarly, Andrei et al. (2016) reported that TEIQue scores accounted for incremental variance in 84.2% of analyses across 18 selected studies.

### Trait Emotional Intelligence as a Predictor of Psychopathology Criteria in Clinical Samples

Petrides et al. (2017) demonstrated the protective role of trait EI in psychopathology on a transdiagnostic clinical sample (i.e., comprising clinical patients with a range of diagnoses). The researchers fitted a model in SEM, in which they included three predictors: trait EI, a mindfulness questionnaire, and a measure of irrational beliefs, reporting that these predictors accounted for 44% of the variance in psychopathology. There were substantial predictive and protective effects from trait EI and mindfulness on irrational beliefs and psychopathology.

Several other syndromes and disorders have been related to trait EI. For instance, Petrides et al. (2011) compared a sample of clinically diagnosed Asperger patients in the United Kingdom with a control sample taken from normative data, using the full form of the TEIQue. The researchers reported a significantly higher global trait EI for the controls than for the clinical sample (*p* < 0.001, η*p*^2^ = 0.40). This trend was fully supported when including the factor-level as predictors (i.e., Wellbeing, Self-control, Emotionality, and Sociability), and partially replicated—with the exceptions of three facets— when testing the same effect after including the fifteen facets that the TEIQue allows.

Furthermore, Aslanidou et al. (2018) reported significantly lower global trait EI and factor-level scores (except the emotionality factor) for individuals suffering from drug addiction when compared to controls. In the aforementioned study, the difference in trait EI means between addicted individuals and controls were of medium effect size for global trait EI, Wellbeing and Sociability, whereas the mean difference regarding Self-control presented a small effect size. In this study, trait EI, and mostly the Wellbeing factor, was negatively and significantly correlated with depression, anxiety, and somatic symptoms (*p* < 0.01, with *R*^2^ of 0.45, 0.16, and 0.18, respectively).

In addition, personality disorders (Sinclair and Feigenbaum, 2012), emotion dysregulation (Petrides et al., 2007b), and psychopathy (Malterer et al., 2008) have been found inversely associated with trait EI. For instance, regarding personality disorders, Sinclair and Feigenbaum (2012) reported that trait EI accurately predicted borderline personality disorder in 95.8% of cases. This effect remained the strongest even after including emotion regulation and mindfulness measures, which did not significantly increase model fit for the prediction. After careful examination of the literature, it is necessary to highlight that there is a dearth of trait EI theory-driven research with true clinical samples, as most studies have focused on global health measures in predominantly healthy populations (Hansen et al., 2009; Zeidner et al., 2012; Petrides et al., 2017). This creates a gap that must be bridged, as clinical samples yield lower trait EI means than those from mainly healthy individuals (Zeidner et al., 2012; Rudenstine and Espinosa, 2018; Espinosa and Rudenstine, 2020; Pérez-Díaz and Petrides, 2021).

## Research Aims

Different populations, cultures, and other sociodemographic and economic peculiarities may affect the interpretation and cross-cultural validity of trait EI in clinical settings. Therefore, the present study had two main aims. First, to provide evidence of measurement invariance concerning the preceding sociodemographic variables with clinical populations. Second, to test for trait EI differences across influential sociodemographic variables (gender, age, educational level, civil status, and occupation) in clinical cross-cultural populations. The study compared datasets from clinical populations in three countries with distinct characteristics (e.g., location, socio-political regime, culture, and economic development), albeit sharing the same language (Spanish). To our knowledge, it is the first attempt to profile clinical populations on trait EI from a cross-cultural perspective and to provide cross-country evidence of measurement equivalence. Trait EI theory is especially appropriate for these aims, as its taxonomy and measurement instruments have strong conceptual and explanatory power to predict attitudes, behaviours, and performance (Petrides et al., 2007c).

## Materials and Methods

### Participants

Data were obtained in Chile, Peru, and Spain from clinical populations. Collectively, the three datasets comprised 528 participants, 23% from Chile (120), 28% from Peru (150), and 49% from Spain (258). Only the Chilean dataset has been employed in previous research (i.e., Pérez-Díaz and Petrides, 2021). Participants did not receive any compensation. The inclusion criteria were as follows: (a) aged 17 years or above and (b) being currently treated for a mental health condition by a qualified mental health provider (i.e., either a psychiatrist or a psychologist). Most participants met the criteria either for any mood or anxiety disorders, which are the most prevalent diagnoses in clinical psychological settings, approximately affecting 8% of the global population (World Health Organization [WHO], 2017). For instance, 31% of the Peruvian participants had suffered from mood disorders, 58% had suffered from anxiety disorders, which combined accounted for 89% of the Peruvian sample, whilst the remaining 11% corresponded to other less frequent mental health disorders (e.g., autism spectrum, eating disorders, and ADHD). Participants diagnosed with a severe disorder/psychopathology (e.g., schizophrenia or severe borderline personality disorders), as diagnosed by the practitioners based on the Diagnostic and Statistical Manual of Mental Disorders (DSM-5) (American Psychiatric Association [APA], 2013), were excluded from the study to protect them from any potential harm arising from the research.

In the pooled dataset, 280 participants were women (53%) and 248 were men (47%). Mean age was 34.38 years, standard deviation [SD] = 11.12, *minimum* = 17, *maximum* = 74). Children and adolescent TEIQues are currently available, although they have not yet been validated in Peru and Chile. Regarding main occupations, most participants worked in the private sector (31%), followed by students (30%) and those in the public sector (19%). The unemployed accounted for 12% of the pooled sample. Participants who declared a non-listed occupation were reached 5% of the pooled sample. Those working in the education sector either as teachers or as lecturers accounted for 3% of participants. This categorisation followed Pérez-Díaz et al. (2021), who compared trait EI means across different clustered occupations: professionals working in the public sector (e.g., health workers, such as physicians, nurses, psychologists, public accountants, public managers, social workers, and military forces), those working in the private sector (e.g., managers, executives, private accountants, entrepreneurs, engineers, and sales personnel), individuals employed in the field of education either as teachers or lecturers, students, the unemployed, and others. Regarding educational attainment, 49% of participants had obtained or were in the process of obtaining a higher education certificate or university degree, 35% had completed secondary education, whilst 16% held a postgraduate qualification (13% Master’s and 3% PhD). Regarding civil status, 51.7% of participants were single, 22.3% were in a relationship, 16.7% were married, 8.9% were divorced or separated, and the remaining 0.4% declared a non-listed civil status. The dataset is available at https://data.mendeley.com/datasets/f23zhjcwcv/2.

### Measures

We used the Chilean-Spanish-TEIQue-SF in Chile and Peru (Pérez-Díaz and Petrides, 2021) and the Spanish translation in Spain (Pérez-González, 2010). These instruments have the same overall layout and number of items (30) as the original English version (TEIQue-SF; Petrides, 2009), with minor linguistic differences. Items are responded to on a 7-point Likert scale, ranging from 1 (Completely Disagree) to 7 (Completely Agree). All surveys included questions on the relevant sociodemographic variables. The instrument was reliable at the global trait EI level in Chile (α = 0.88, Pérez-Díaz and Petrides, 2021) and Spain (α = 0.85, Pérez-González, 2010), although with lower alphas at the factor level (0.61–0.87), especially for Emotionality and Sociability. The four-factor structure has been replicated in Chile (Pérez-Díaz and Petrides, 2021) and Spain (Laborde et al., 2016), although there have been no previous applications in Peru.

### Design and Procedure

Chilean and Spanish participants completed (either individually or collectively) the paper-and-pencil version of the TEIQue-SF, whilst Peruvian participants responded online due to Coronavirus Disease (COVID) restrictions. Pertinent local ethics boards approved all phases of the data collection. In Chile, the Ethics Committee of the University College London (UCL) granted ethical approval with project ID: 12971/00, as the data were originally collected as part of doctoral research (Perez Diaz, 2021). In Spain, the Ethical Board of the University of Cadiz, Spain approved the study. In Peru, the Psychology Research Institute of Universidad San Martín de Porres provided ethics clearance.

### Data Analysis Plan

We first determined that the observations followed the multivariate normal distribution for global trait EI and that the assumption of homogeneity of variances was met for all the sociodemographic variables, as assessed by Levene’s statistic. We conducted analysis of covariance (ANCOVA) and reported Eta Squared (η^2^) as a measure of effect size. Regarding *post hoc* analyses, we conducted multiple comparisons through the Bonferroni correction to control the Type I error. We tested for three kinds of measurement invariances: configural, metric, and scalar (Putnick and Bornstein, 2016), following the recommendations of Hu and Bentler (1999), Cheung and Rensvold (2002), and Meade et al. (2008).

**FIGURE 1:**
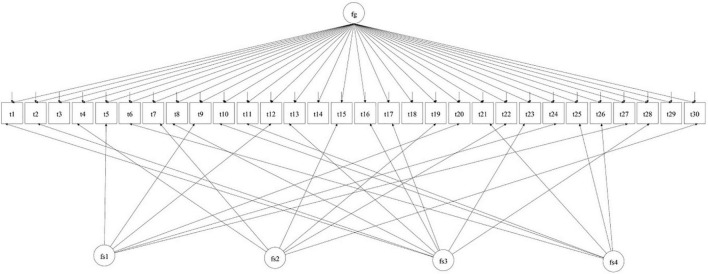
Illustration of the base ESEM bi-factor model tested through measurement invariance analyses across the chosen sociodemographic variables. Fg stands for global trait EI, fsl for well-being, fs2 for self-control, fs3 for emotionality and fs4 for sociability. The TEIQue-SF items are t1 to t30. From “Invariance of the trait emotional intelligence construct across populations and sociodemographic variables” by Pérez-Díaz et al. (2021). Copyright 2021 by the International Society for the Study of Individual Differences (ISSID).

We evaluated model fit through maximum likelihood with robust standard errors (MLR) estimations following Meade et al. (2008). For testing measurement invariance, we treated the age variable as categorical, which is a common practice (Millsap, 2011) that returns equivalent results to those directly obtained from conditional probability data (Muthén and Asparouhov, 2002). Accordingly, we dichotomised the age variable at the 50th percentile of its distribution (i.e., 30 years) and labelled these groups as Younger (17–30) and Older (31–80). In all measurement invariance analyses, we started with a basic bifactor Exploratory Structural Equation Model (ESEM) because it has proved suitable in previous research (see Pérez-Díaz and Petrides, 2021; Pérez-Díaz et al., 2021). ESEM and MGESEM (Multigroup ESEM) are special cases of SEM (i.e., Structural Equation Model, Asparohov and Muthén, 2009). This model is depicted in Figure 1.

## Results

### Multiple Imputations of the Data

We implemented multiple imputations by chained equations for treating missing values with the R package Multivariate Imputation *via* Chained Equations (MICE) (Van Buuren and Groothuis-Oudshoorn, 2011). Most of the TEIQue-SF items in the datasets were complete, with less than0.3% of missing values. Similarly, all the sociodemographic variables had less than 1% of missing values. Therefore, we performed five imputations and conducted all further statistical analyses with the pooled imputed dataset.

### Reliability Analyses

The global trait EI score was highly reliable in the pooled dataset (ω = 0.92, α = 0.89). The proportion of scale variance due to the general factor (global trait EI) only, as estimated by ω*h*, was 68%. In addition, trait EI factors mainly showed adequate-to-high reliability (Wellbeing: ω = 0.85, α = 0.85; Self-control: ω = 0.85, α = 0.69; Emotionality: ω = 0.60, α = 0.68; and Sociability: ω = 0.68, α = 0.64); although the values were considerably lower than that of the global trait EI score, which the TEIQue-SF was specifically designed to measure. Moreover, lower than desired (i.e., < 0.70) reliability scores at the factor level (most noticeably for Emotionality and Sociability) had been previously reported in clinical TEIQue-SF samples (Petrides et al., 2017; Jacobs et al., 2021; Perez Diaz, 2021).

### Measurement Invariance

The measurement invariance analyses revealed that the trait EI factor structure, as measured by the TEIQue-SF and modelled bifactorially, was invariant up to the scalar (latent means) level for gender and education. The comparative fir index (CFI) reached the 0.9 cutoff threshold, with CFI changes across nested models falling below the 0.01 cutoff criterion recommended by Cheung and Rensvold (2002).

Moreover, the root mean square error of approximations (RMSEAs) and standardised root mean residuals (SRMRs) in all modelled measurement invariance analyses were below their respective recommended thresholds (i.e., 0.06 and 0.08; Hu and Bentler, 1999). Similarly, changes in the RMSEA and SRMR were in the recommended range (i.e., ≤ 0.015 and ≤ 0.030, respectively).

Regarding age, although RMSEA and SRMR changes across the measurement invariance phases (configural, metric, and scalar) were within the expected boundaries, CFI changes were not, suggesting that the instrument may be non-invariant in clinical populations of different ages, at least in the countries examined in the present study. Concerning civil status, the analyses revealed non-invariance at the scalar step (Δ*CFI* > 0.01). Regarding occupation, CFI was below the threshold of 0.9 for configural invariance, which prevented progressing to the metric and scalar steps of the process (e.g., Hu and Bentler, 1999), even though all remaining fit indices were on the edge of the expected boundaries. Detailed results from these analyses are depicted in Table 1.

**TABLE 1:** Multiple group measurement invariance comparisons by sociodemographic characteristics.

Models	χ^2^	Δχ^2^	*df*	*CFI*	Δ*CFI*	*RMSEA*	Δ *RMSEA*	*RMSEALb*	*RMSEAUb*	*SRMR*	Δ *SRMR*
1. Gender											
Configural	1070.90	–	703	0.917	–	0.045	–	0.039	0.050	0.049	–
Metric	1120.81	49.91	733	0.913	0.004	0.045	0.000	0.039	0.050	0.052	0.003
Scalar	1099.95	20.86	728	0.916	0.003	0.044	0.001	0.039	0.049	0.051	0.001
2. Age											
Configural	1085.76	–	586	0.887	–	0.057	–	0.052	0.062	0.040	–
Metric	1164.16	78.40	737	0.904	0.017	0.047	0.010	0.042	0.052	0.051	0.011
Scalar	1323.80	159.64	732	0.866	0.028	0.055	0.008	0.051	0.060	0.051	0.000
3. Education											
Configural	1576.51	–	1114	0.901	–	0.049	–	0.043	0.054	0.061	–
Metric	1664.21	87.70	1174	0.896	0.005	0.049	0.000	0.043	0.054	0.065	0.004
Scalar	1646.01	18.20	1164	0.897	0.001	0.049	0.000	0.043	0.054	0.062	0.003
4. Civil status											
Configural	1642.57	–	1117	0.884	–	0.054	–	0.049	0.060	0.063	–
Metric	1861.72	122.09	1192	0.894	0.010	0.051	0.001	0.047	0.056	0.067	0.012
Scalar	1755.58	106.14	1180	0.873	0.019	0.055	0.004	0.050	0.061	0.069	0.002
5. Occupation											
Configural	1713.73	–	1123	0.861	–	0.060	–	0.054	0.066	0.067	–
Metric	1798.55	84.82	1183	0.855	0.006	0.060	0.000	0.054	0.065	0.072	0.005
Scalar	1792.44	6.11	1173	0.854	0.001	0.060	0.000	0.055	0.066	0.069	0.003

*Model 1 = gender, N = 528, nWomen = 280, nMen = 248. Model 2 = age, N = 528, nYoung = 230, nSenior = 298. Model 3 = education, N = 528, nSecondary = 187, nUniversity = 257, nGraduate = 84. Model 4 = civil status, N = 479, nSingle = 273, nRelationship = 118, nMarried = 88. Model 5 = occupation, N = 438, nPrivate = 164, nPublic = 114, nStudent = 160. χ^2^ = chi squared, Δχ^2^ = chi squared difference, df = degrees of freedom, CFI = comparative fit index, ΔCFI = CFI difference, RMSEA = root mean square error of approximation, Δ RMSEA = RMSEA difference, RMSEALb = RMSEA lower bound, RMSEAUb = RMSEA upper bound. SRMR = standardised root mean residual, Δ SRMR = SRMR difference.*

### Trait Emotional Intelligence Means Differences Across Sociodemographic Variables

Descriptive statistics for the trait EI variables in each country are depicted in Table 2. The Peruvian dataset showed the highest global trait EI mean from the three datasets, followed by the Chilean and the Spanish samples. We first assessed if parametric requirements were met through the Kolmogorov-Smirnov-Lilliefors (KSL) test of normality and the Levene’s test of equality of error variances. The test of normality (*D)* showed that global trait EI was normally distributed in all three datasets. Moreover, global trait EI variances across the three-country datasets were homogenous according to the Levene’s test [*F* = 2.25 (2,525), *p* = 0.107].

**TABLE 2:** Descriptive statistics for the TEIQue-SF datasets.

Trait EI measure	*Min*	*Max*	*M*	*Me*	*SD*	*IQR*	*Skew*	*Kurt*
**1. Full cross-cultural dataset**								
Global trait EI	1.83	6.80	4.38	4.40	0.98	1.43	−0.06	−0.45
Well-being	1.00	7.00	4.58	4.67	1.38	2.17	−0.28	−0.66
Self-control	1.00	7.00	3.96	4.00	1.19	1.67	0.06	−0.35
Emotionality	1.00	7.00	4.66	4.63	1.06	1.38	−0.27	−0.27
Sociability	1.33	7.00	4.32	4.33	1.11	1.50	−0.25	−0.17
**2. Chile**								
Global trait EI	2.63	6.80	4.75	4.80	0.84	1.26	−0.15	−0.26
Well-being	1.50	7.00	5.05	5.33	1.25	1.79	−0.50	−0.47
Self-control	2.00	6.83	4.39	4.33	1.17	1.83	0.15	−0.70
Emotionality	2.13	6.88	4.88	5.13	0.99	1.47	−0.35	−0.37
Sociability	1.33	6.33	4.63	4.67	0.88	1.33	−0.61	1.24
**3. Peru**								
Global trait EI	2.13	6.80	4.56	4.53	1.00	1.58	−0.05	−0.49
Well-being	1.83	7.00	4.87	5.00	1.29	2.21	−0.26	−0.83
Self-control	1.33	7.00	4.08	4.00	1.18	1.50	0.13	−0.23
Emotionality	1.00	7.00	4.66	4.63	1.09	1.53	−0.36	0.03
Sociability	2.17	7.00	4.68	4.67	1.02	1.50	−0.07	−0.41
**4. Spain**								
Global trait EI	1.83	6.33	4.11	4.07	0.94	1.38	0.01	−0.49
Well-being	1.00	7.00	4.19	4.33	1.39	2.17	−0.14	−0.66
Self-control	1.00	6.50	3.69	3.75	1.13	1.67	−0.07	−0.53
Emotionality	1.88	6.75	4.56	4.63	1.06	1.41	−0.16	−0.38
Sociability	1.50	6.83	3.97	4.00	1.15	1.50	−0.03	−0.36

*1. Full cross-cultural dataset, N = 528. 2. Chile, n = 120. 3. Peru, n = 150. 4. Spain, n = 258. All descriptive statistics refer to the pooled imputed dataset. EI = emotional intelligence. Min = minimum, Max = maximum, M = mean, Me = median, SD = standard deviation, IQR = interquartile range, Skew = skewness, Kurt = kurtosis.*

At the factor level of trait EI, in Chile, Wellbeing and Emotionality were deviated from normality [*D*(120) = 0.12, *p* < 0.001; *D*(120) = 0.11, *p* = 0.002], whereas Self-control and Sociability were both normally distributed (*p* = 0.200). In Peru, Self-control and Sociability were normally distributed (*p* = 0.093, *p* = 0.082), whereas Wellbeing and Emotionality were deviated from normality [*D*(150) = 0.08, *p* = 0.018; *D*(150) = 0.07, *p* = 0.041]. In Spain, Emotionality was normally distributed (*p* = 0.200), whereas Wellbeing, Self-control, and Sociability were deviated from the expected distribution [*D*(317) = 0.07, *p* = 0.004; *D*(317) = 0.06, *p* = 0.017, *D*(317) = 0.06, *p* = 0.037]. After visual inspection of quantile-quantile (q-q) plots from the pooled dataset, histograms, and the KSL statistic, we concluded that the deviation from normality at the factor level was not substantial. Moreover, the variance was homogenous for three of the four trait EI factors in the pooled dataset: {Wellbeing [*F* = 1.58 (2,525), *p* = 0.206], Self-control [*F* = 0.18 (2,525), *p* = 0.838], Emotionality [*F* = 0.29 (2,525), *p* = 0.749], with Sociability displaying heterogeneity of variances [*F* = 6.06 (2,525), *p* = 0.003]}.

We conducted ANCOVAs with the sociodemographic variables as independent variables, age as covariate and trait EI (global + four factors) as the dependent variables, the homogeneity of variance assumption was met in all cases {global trait EI [*F* = 0.99 (143,384), *p* = 0.509]; Wellbeing [*F* = 1.02 (143,384), *p* = 0.448]; Self-control [*F* = 0.92 (143,384), *p* = 0.718]; Emotionality [*F* = 0.97 (143,384), *p* = 0.580]; and Sociability [*F* = 0.93 (143,384), *p* = 0.706]}. These analyses revealed that country explained significant trait EI variance over and above the other IVs in the main effects model [global trait EI, *F* (2) = 20.79, *p* < 0.001, η*^2^* = 0.09; Wellbeing, *F* (2) = 23.68, *p* < 0.001, η*^2^* = 0.08; Self-control, *F* (2) = 19.44, *p* < 0.001, η*^2^* = 0.07; Emotionality, *F* (2) = 3.76, *p* = 0.024, η*^2^* = 0.01; and Sociability, *F* (2) = 25.28, *p* < 0.001, η*^2^* = 0.09]. In addition, in these analyses, age had a significant main effect on Self-control [*F* (1) = 4.23, *p* = 0.040, η*^2^* = 0.01], occupation had a significant main effect on Emotionality [*F* (3) = 2.72, *p* = 0.044, η*^2^* = 0.02], and education had a significant main effect on Sociability [*F* (2) = 5.38, *p* = 0.001, η*^2^* = 0.02]. Last, gender had a significant main effect on Self-control [*F* (1) = 3.96, *p* = 0.047, η*^2^* = 0.01] and on Emotionality [*F* (1) = 5.72, *p* = 0.017, η*^2^* = 0.01]. All these main effects were small in size (i.e., η*^2^* between 0.1 and 0.5).

There were significant two-way interactions between country and occupation on global trait EI [*F* (6) = 2.35, *p* = 0.030, η*^2^* = 0.03], and between gender and occupation [*F* (6) = 2.83, *p* = 0.038, η*^2^* = 0.02] on global trait EI. Gender and country had a significant interaction Self-control [*F* (6) = 4.96, *p* = 0.007, η*^2^* = 0.02], whilst country and occupation had significant interactions on Self-control [*F* (6) = 2.21, *p* = 0.041, η*^2^* = 0.03] and on Emotionality [*F* (6) = 2.22, *p* = 0.040, η*^2^* = 0.03]. Finally, gender and occupation had a significant interaction on Sociability [*F* (6) = 2.91, *p* = 0.034, η*^2^* = 0.02]. With respect to three-way interactions, the assumption of homogeneity of variances was violated for all trait EI-dependent variables except Emotionality. In addition, there was a high number of parameters in these models, which reduces statistical power. For these reasons, we did not proceed further with these analyses.

Chilean participants scored significantly higher than Spaniards on global trait EI (*p* < 0.001, η*^2^* = 0.09), Wellbeing (*p* < 0.001, η*^2^* = 0.08), Self-control (*p* < 0.001, η*^2^* = 0.07), Emotionality (*p* = 0.021, η*^2^* = 0.01), and Sociability (*p* < 0.001, η*^2^* = 0.09). Chilean participants also scored higher than Peruvians on Self-control (*p* = 0.024, η*^2^* = 0.07). Regarding education, participants with graduate education (Master’s degree or PhD) scored significantly higher than those with secondary education (*p* = 0.004, η*^2^* = 0.09) on Sociability. Regarding occupation, students scored significantly higher than participants working in the private sector (*p* = 0.046, η*^2^* = 0.02). Men scored marginally higher than women (*p* = 0.047, η*^2^* = 0.01) on Self-control, whereas women scored higher on Emotionality (*p* = 0.017, η*^2^* = 0.01). There were no differences in civil status. Effects sizes were generally medium-to-small.

## Discussion

The present study represents the first cross-cultural inquiry into the role of trait EI in clinical populations. The research portrays rather a typical trait EI means from clients receiving mental healthcare in each of the studied countries, as trait EI has consistently been found lowered across various clinical conditions (Zeidner et al., 2012), and more cross-cultural resembles than differences were revealed in our research. Moreover, similar global trait EI means have been reported with relatively large clinical samples (i.e., 200 participants), as this score fell between 4.28 and 4.53 (see Rudenstine and Espinosa, 2018; Espinosa and Rudenstine, 2020; Jacobs et al., 2021), which is congruent with the global trait EI mean of the pooled cross-cultural dataset hereby presented (4.38), a trend that also replicates at the factor-level of trait EI in these studies (Except for Rudenstine and Espinosa, 2018 that did not report factor-level scores), and that deviates from the reported cross-cultural mean of global trait EI in general populations (i.e., 4.85, Pérez-Díaz et al., 2021). Therefore, the results support the premise that cultural nuisances in each of the countries affect the distribution of trait EI in clinical participants from different sociodemographic strata, albeit Chileans and Peruvians participants resembled each other more than Spaniards, and that clinical trait EI means are consistently lower than those obtained in general samples.

Regarding measurement invariance, the analyses support strong (i.e., scalar) cross-cultural invariance of trait EI (as measured by the TEIQue-SF) concerning gender and education. The main advantages of the chosen approach in comparison to extant related research (e.g., Tsaousis and Kazi, 2013; Siegling et al., 2015a) are threefold. First, the implementation of a multidimensional baseline model, which included both the global and the factor-level of the construct, whereas former research had modelled either a global score or factor scores exclusively. Second, the richness of the datasets with participants from three different countries and variants of Spanish. Third, the strategy of testing for trait EI invariance beyond gender and age, which is comparable with related research on trait EI across countries and sociodemographic strata in community samples from Chile, Brazil, and Italy (see Pérez-Díaz et al., 2021).

Our findings were consistent with previous trait EI examinations in clinical transdiagnostic participants (i.e., carrying different diagnoses; Andrei and Petrides, 2013; Petrides et al., 2017; Perez Diaz, 2021) and meta-analytical research with clinical correlates (Sarrionandia and Mikolajczak, 2020). Trait EI was unequally distributed across the layers of the studied sociodemographic variables, due to participants with higher educational attainment were generally more emotionally and socially effective than their less-educated peers, as students were when compared to those employed in the private sector. However, it should be noted that trait EI investigations with clinical participants are scarce, and thus, most informed health outcomes, in which the predictive role of trait EI was tested, were obtained from community samples (e.g., students).

The present findings corroborated the previously reported trend of limited gender differences on global trait EI in with clinical population (e.g., Pérez-Díaz and Petrides, 2021), as male-favouring differences of small effect size were found on the Self-control factor in Peru and Spain, and female-favouring differences were found in Emotionality. The overall conclusion seems to be that a small effect size usually accompanies any trait EI difference of means across genders in meta-analytical research (e.g., MacCann et al., 2020).

The effects of other sociodemographic correlates, such as educational attainment, civil status, and occupational status on trait EI, have not been extensively investigated in the literature since participants are typically described and controlled for the effects of gender and age (Kun and Demetrovics, 2010). However, some similarities arise regarding the role of sociodemographic variables on relevant clinical criteria with participants from mental health settings (i.e., clinical). For instance, Rudenstine and Espinosa (2018) reported that even though gender and age did not significantly explain the variance of psychological distress (i.e., depression, anxiety, and somatisation), whereas income and educational attainment did, as these variables correlated negatively with these three symptoms.

In related research (Espinosa and Rudenstine, 2020), the same researchers corroborated that education and age were negatively correlated to several clinical criteria. However, in this piece of research, women scored higher than men on one clinical criterion (i.e., phobic anxiety), and a negative association was reported between age and several symptoms, meaning that older participants experienced less psychological distress whilst younger participants reported more. In this regard, both income and education acted as protective factors against psychopathology, similar to what our present findings inform on trait EI with highly educated peers (graduate degrees) and those occupied either as students or in the public sector compared to those in other occupational strata.

These findings pose a challenge on the potential confounding variables affecting trait EI (e.g., socioeconomic status) and provide a basis for understanding trait EI differences across the levels of the abovementioned sociodemographic variables. Concerning occupation, although there is a dearth of evidence on the role of this variable to trait EI with clinical populations, the literature with general populations highlights that some professions, especially those from the public sector (e.g., health practitioners, government) or exerting leadership and mentorship roles (e.g., education, management) usually have higher levels of trait EI (Siegling et al., 2014; Li et al., 2018; Pérez-Díaz et al., 2021). The results of the present study are consistent with these findings, as can be observed by the positive predictive role that both occupation and education, respectively, had on Emotionality and Sociability.

The ANCOVA analyses revealed higher homogeneity between the Chilean and Peruvian datasets, which is expectable as bordering countries tend to display similar means on personality measures (Allik and McCrae, 2004), such as trait EI. In contrast, according to Allik and McCrae, regions either geographically or historically apart differentiate more on personality trait scores, which may explain why the Chilean and Peruvian trait EI means resemble more each other compared to the Spanish (as depicted in Table 2), and the discrete interaction between country and occupation. Moreover, McCrae (2002) has reported that European cultures showed a higher dispersion than North American and Asian cultures on the domains of the five-factor model of personality (FFM), having Spaniards the fifth-highest standard deviation across 36 countries included in the study. We argue that a similar trend could take place on trait EI, although further cross-cultural research is necessary to clarify this.

The present study supported mean equivalence (i.e., scalar invariance) of trait EI across clinical populations from different countries and the studied sociodemographic variables of gender and education, being this the first attempt in the literature, a task of major methodological interest (Byrne and Campbell, 1999). As a limitation of our study, it is important to recognise that categorising educational attainment, civil status, and occupation in cross-cultural research is methodologically more challenging than sorting gender and age, as the former variables are highly dependent on local cultural nuisances. A shred of evidence exemplifying this was that the main effects of gender and occupation explained significant variance and that each of these variables interacted with the country on trait EI in the ANCOVA analyses. Further research should test the invariance of the hereby reported non-invariant sociodemographic variables on trait EI and modelling other sociodemographic correlates that allow expanding the nomological matrix in which the trait EI construct deploys across clinical populations, with higher sample size and more heterogeneous populations.

## Conclusion

Overall, the findings highlight the cross-cultural stability and validity of the trait EI construct across clinical populations, as measured by the TEIQue-SF, irrespective of cultural, socioeconomic, and linguistic correlates. This gives the TEIQue-SF potential value as a complementary clinical diagnostic tool of common application in Latin American countries, filling a gap in the screening of emotional vulnerability in transdiagnostic disorders, where the underlying basis is often related to emotional difficulties or emotional dysregulation (e.g., Muñoz-Navarro et al., 2022; Zarate-Guerrero et al., 2022). However, a word of caution is necessary regarding the non-invariance discovered in age, civil status, and occupations across the examined countries. To the best of our knowledge, this is the first cross-cultural examination on trait EI in clinical populations, as conceptualised by Petrides and colleagues.

## Data Availability Statement

The datasets presented in this study can be found in online repositories. The names of the repository/repositories and accession number(s) can be found below: https://data.mendeley.com/datasets/f23zhjcwcv/2.

## Ethics Statement

In Chile, the University College London (UCL) - Ethics Committee granted ethical approval with project ID: 12971/00, as the data were originally collected in Chile as a part of doctoral research (Pérez-Díaz et al., 2021). In Spain, the Ethical Board of the University of Cadiz, Spain, approved the study. In Peru, the Psychology Research Institute of Universidad San Martín de Porres provided ethics clearance. The patients/participants provided their written informed consent to participate in this study.

## Author Contributions

PP-D and KP contributed to conception and design of the study. PP-D, DM-M, and MG-G organised the database. PP-D performed the statistical analysis and wrote the first draft of the manuscript. PP-D, KP, and J-CP-G wrote sections of the manuscript. PP-D, MV-S, RM-R, NF-R, DM-M, and MG-G contributed with data collection. All authors contributed to manuscript revision, read, and approved the submission.

## Conflict of Interest

The authors declare that the research was conducted in the absence of any commercial or financial relationships that could be construed as a potential conflict of interest.

## Publisher’s Note

All claims expressed in this article are solely those of the authors and do not necessarily represent those of their affiliated organizations, or those of the publisher, the editors and the reviewers. Any product that may be evaluated in this article, or claim that may be made by its manufacturer, is not guaranteed or endorsed by the publisher.
